# Hydrology and hydrological extremes under climate change scenarios in the Bosque watershed, North-Central Texas, USA

**DOI:** 10.1007/s11356-023-27477-1

**Published:** 2023-05-18

**Authors:** Gebrekidan Worku Tefera, Ram Lakhan Ray

**Affiliations:** Cooperative Agricultural Research Center, College of Agriculture and Human Sciences, Prairie View A&M University, Prairie View, TX 77446 USA

**Keywords:** Climate change scenarios, Downscaling techniques, High flow, Low flow, Texas, SWAT

## Abstract

This study evaluates hydrology and hydrological extremes under future climate change scenarios. The climate change scenarios were developed from multiple Global Circulation Models (GCMs), Representative Concentration Pathway (RCP) scenarios, and statistical downscaling techniques. To ensure hydrological model robustness, the Soil Water Assessment Tool (SWAT) was calibrated and validated using the Differential Split Sample Test (DSST) approach. The model was also calibrated and validated at the multi-gauges of the watershed. Future climate change scenarios revealed a reduction in precipitation (in the order of −9.1% to 4.9%) and a consistent increase in maximum temperature (0.34°C to 4.10°C) and minimum temperature (−0.15 °C to 3.7°C) in different climate model simulations. The climate change scenarios triggered a reduction of surface runoff and streamflow and a moderate increase in evapotranspiration. Future climate change scenarios projected a decrease in high flow (Q5) and low flow (Q95). A higher reduction of Q5 and annual minimum flow is also simulated in future climate scenarios, whereas an increase in annual maximum flow is simulated in climate change scenarios developed from the RCP8.5 emission scenario. The study suggests optimal water management structures which can reduce the effect of change in high and low flows.

## Introduction

Climate change has a non-consistent effect on surface runoff and streamflow among different regions of the world. In the Midwestern, Northwestern, and Northeastern USA, an increase in streamflow was observed as a response to climate change, whereas a reduction in streamflow was observed in the southern states of the USA (Romero-Lankao et al., 2014; Talib and Randhir, 2017). For instance, in the Texas High Plains, prolonged high temperature and low rainfall caused severe drought events in 2011 and 2012 (Hoerling et al., 2013; Ray et al., 2018, 2017). Climate change also triggers a change in the seasonal magnitude and timing of streamflow. In snow-dominated watersheds, an increase in temperature has resulted in a shift in the magnitude and timing of hydrological events (Bates et al., 2008; Kim et al., 2017; Ray et al., 2016; Romero-Lankao et al., 2014). In the snow-dominated rivers of western North America, snowmelt attributed to climate change triggers an early peak flow of runoff (Barnett et al., 2005; Das et al., 2011). For instance, in the Ohio-Tennessee River Basin, an increase in early peak flow of runoff, i.e., the highest runoff in February and March and a reduced runoff during June–September, was simulated using different climate model simulations (Panagopoulos et al., 2015). These indicate that region and watershed-specific studies of climate change’s effect on hydrological characteristics are essential.

The effect of climate change on hydrological extremes also warrants special focus since extreme values are sensitive to climate change and could be impacted by uncertainties in climate modeling. Climate change amplifies low and high-flow change signals mainly attributed to changes in precipitation and temperature (De Girolamo et al., 2022; Kay et al., 2021; Marx et al., 2018). For instance, in 10 gauges of the Brazos River Basin of Texas, a higher reduction in minimum flow than maximum flow was observed from 1955 to 2014 (Sohoulande Djebou, 2017). Alas, changes in hydrological extreme events trigger more profound effects on the natural and anthropogenic ecosystems than changes in annual, seasonal, and monthly scales (Arnell, 2004; Taye and Willems, 2012). The 2015 record-breaking flood event has caused an estimated $2.6 billion damage in Texas and Oklahoma (USGCRP, 2018). Extreme hydrological events such as high flow frequency, extreme peak flow quantile, and extreme low flow quantile are characterized by uncertainty due to the uncertainties derived from climate data (Kay et al., 2021; Meresa et al., 2021). For instance, high uncertainty in precipitation of climate model simulation has triggered uncertainty in hydrological and extreme hydrological values in the Southeast Asian basins (Shrestha et al., 2021). Thus, caution is needed to maintain the quality of climate data before using it for hydrological modeling.

Hydrological climate change impact assessment studies are prone to have considerable uncertainties attributed to greenhouse gas emission scenarios, climate models, downscaling and bias correction techniques, and hydrological models (IPCC, 2013; Kundzewicz et al., 2018). For this, it is non-trivial to use the output of robust climate change scenarios for climate change impact studies. Climate change scenarios are consistent and plausible representations of future climate conditions through blending emission scenarios, climate model simulations, and downscaling techniques (IPCC-TGICA, 2007; IPCC, 2013; Moss et al., 2010). In climate change scenarios, GCMs have limitations in simulating regional and local scale precipitation and cloud cover of the mountain and coastal regions due to low spatial resolutions and inadequate parametrization of regional scale drivers of climate (Flato et al., 2013). For this, it is commendable to use the ensemble of GCM simulations than the single GCM simulation to reduce possible uncertainties stem from multiple sources (Flato et al., 2013; IPCC-TGICA, 2007; Teutschbein and Seibert, 2010). Besides, GCM downscaling is a commonly used technique in climate research to improve the horizontal resolution of climate models, reduce biases in GCM simulations, and better parametrize the effect of factors such as topography on local and regional scale climate (Flato et al., 2013; Fowler et al., 2007; IPCC, 2021).

Dynamical and statistical downscaling techniques involve transferring climate information from GCM simulations to regional and local scales (Flato et al., 2013; IPCC, 2013). In the dynamical downscaling technique, higher resolution climate models, i.e., Regional Climate Models (RCMs), are embedded within a GCM (Flato et al., 2013). However, this technique may also inherit biases from driving GCM, which is used as a boundary condition (Adachi and Tomita, 2020; Fowler et al., 2007). The statistical downscaling technique comprises different methods which develop empirical relationships between raw climate model simulations (RCM and GCM) and local observed climate data (Flato et al., 2013; Fowler et al., 2007; Lanzante et al., 2019). Weather typing schemes, linear methods, and weather generators are common methods of statistical downscaling (Fowler et al., 2007; Hernanz et al., 2022; Lanzante et al., 2020). It is also common to combine and interchangeably use statistical downscaling and statistical bias correction (Cannon et al., 2015; Pourmokhtarian et al., 2016; Wootten et al., 2021). For instance, the cumulative distribution function (CDFt), which adjusts the statistical distributions of climate model simulations, was considered a statistical downscaling technique (Lanzante et al., 2019) and a statistical bias correction technique (Teutschbein and Seibert, 2012). Wootten et al. (2021) have used the ratio delta method and equi-ratio quantile mapping, described as statistical downscaling and bias correction techniques.

The statistical downscaling and bias correction techniques are essential in climate change scenario development to transfer GCM and RCM simulations to smaller spatial scales, reducing climate model simulation biases and adding value to hydrological impact assessment. For instance, surface runoff simulated using bias-corrected RCM simulations is more reliable than surface runoff simulated using raw RCM simulation (Hagemann et al., 2011; Muerth et al., 2013). In five mesoscale catchments in Sweden, streamflow simulations with statistical bias correction were better fitted with the observed streamflow than the simulations without statistical bias adjustment of climate model simulations (Teutschbein and Seibert, 2012). Statistical downscaling and bias correction also add value in reproducing extreme hydro-climatic values than raw climate model simulations (Chen et al., 2013; Ji et al., 2020; Teutschbein and Seibert, 2012; Worku et al., 2020). Multiple statistical downscaling and bias correction techniques may capture the biases from RCM parametrization schemes and bias correction algorithms (Teutschbein and Seibert, 2012; Pourmokhtarian et al., 2016).

Besides, robust hydrological models are essential to reduce uncertainties and develop climate change impact assessment (Baldassarre et al., 2011; Kundzewicz et al., 2018). Identifying sensitive hydrological parameters, optimization algorithms, input data, and best performance measures is essential to reduce uncertainties in hydrological models used for climate impact assessment (Bárdossy and Singh, 2008; Gan et al., 2018). Further, calibration and validation at different temporal scales and areas of the basin (considering hydrological signatures in calibration and validation) are important to reduce the uncertainty of hydrological projections (Huang et al., 2020; Melišová et al., 2020). The conceptual and parameterization structure of hydrological models could also trigger uncertainty in projected hydrological components (Poulin et al., 2011). Thus, caution is needed in selecting, calibrating, and validating hydrological models before using them for climate change impact assessment.

This study was conducted in the Bosque watershed of the Brazos River Basin, Northcentral Texas, where climate change already poses a negative impact on the natural ecosystem and water availability (Hoerling et al., 2013; Shafer et al., 2014). Unlike other studies, this study applies both Differential Split Sample Test (DSST) and multi-site calibration and validation approaches. The DSST was used to test the hydrological model’s capability under changing and even contrasted climate conditions (Daggupati et al., 2015; Guilpart et al., 2020; Huang et al., 2020; Klemeš, 1986). Besides, this calibration and validation approach tests the stationarity assumption where the hydrological models calibrated using observed data are used for future hydrological climate change impact studies assuming the hydrological parameters are unchanging for future climate conditions. Further, the multi-site calibration and validation are to examine non-uniqueness (Beven, 2006) of the hydrological model calibrated at one of the gauges of the watershed.

The objectives of this study are to (1) evaluate the changes in precipitation, temperature, surface runoff, evapotranspiration, and streamflow under future climate change scenarios and (2) examine the magnitude of change in hydrological extremes under future climate change scenarios. This study blends multiple emission scenarios, GCM simulations, statistical downscaling, and bias correction techniques and uses a robustly calibrated and validated hydrological model to reduce uncertainty. This study can be essential to develop optimal water management and agriculture systems that help stakeholders to ensure sustainable water development and agricultural production.

## Materials and method

This study integrates climate change scenarios with hydrological modeling. First, the study develops future climate change scenarios which blend multiple GCMs, emission scenarios, and statistical downscaling techniques. The climate change scenarios were used to analyze changes in precipitation and temperature and as input for the hydrological model. Hydrological model setup, calibration and validation, and uncertainty analysis were conducted before hydrological climate change impact assessment. Finally, the outputs of hydrological model simulations under different climate change scenarios were used to estimate changes in hydrological extremes.

### Study area

**Fig. 1:**
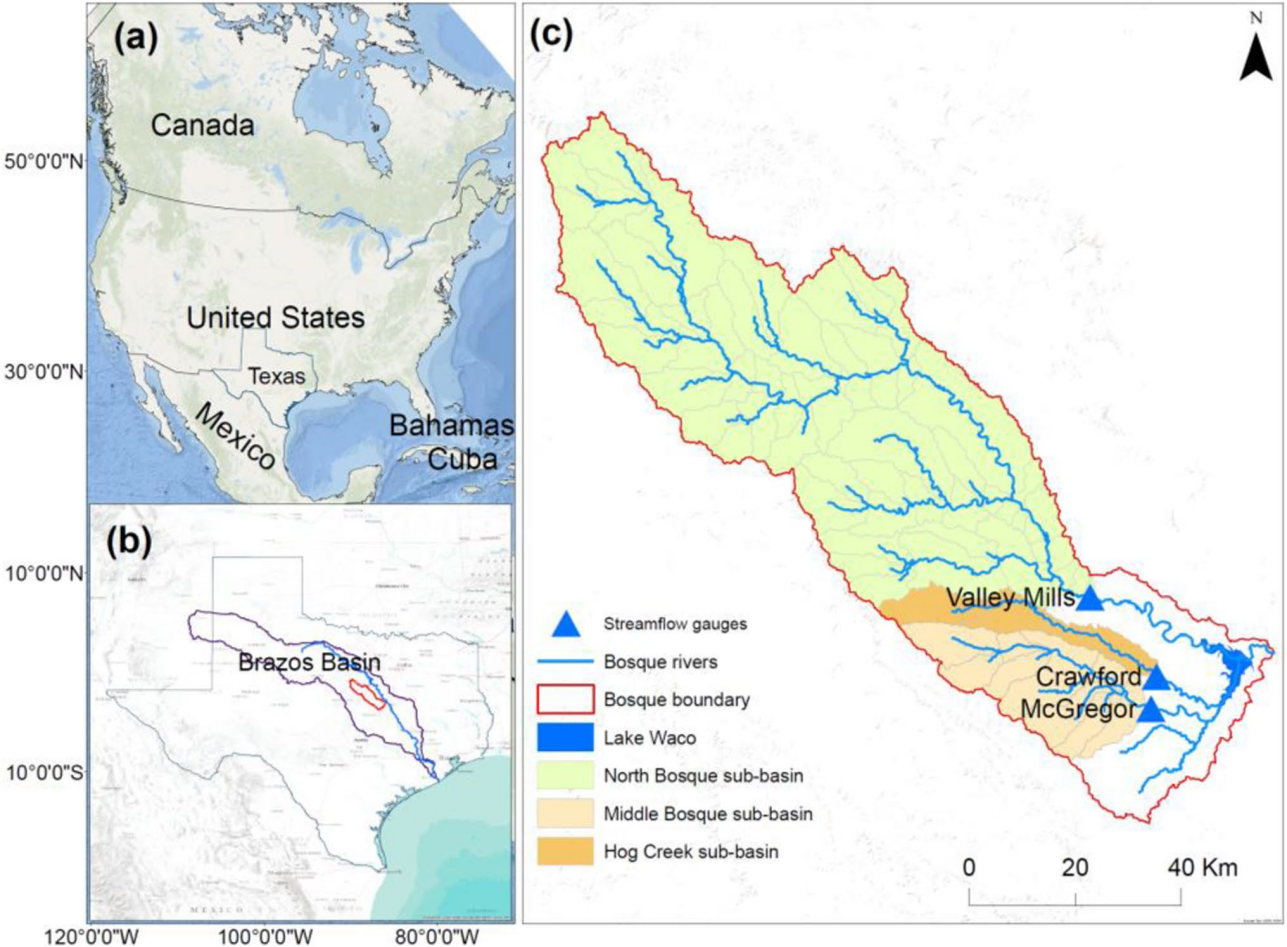
Map of the study area. **a** Location of the USA, **b** location map of Texas and the Brazos River basin, and **c** map of the Bosque watershed and stream discharge stations

The Bosque watershed is located in the Brazos River Basin, which covers most climate zones of Texas (Fig. 1). The watershed has an area of 4300 km^2^. The elevation in the watershed ranges from 111 to 596 meters (Ray et al., 2022). River Bosque drains into Lake Waco and supplies drinking water for a large population of the Waco area. The area of the Bosque watershed is under rangeland, woodland, forage fields, and dairy waste application fields. Dairy production and other agricultural enterprises, including peanut, range-fed cattle, pecan, peach, and forage hay production, are the dominant agricultural activity (Saleh and Gallego, 2007). The major soil types in the watershed include fine sandy loams with sandy clay (hydrologic group C), calcareous clays and clay loams (hydrologic group D), and fine-loamy, siliceous, thermic Udic Paleustalfs (fine-loamy, siliceous, thermic Udic Paleustalfs) The middle part of the Bosque watershed is characterized by deep-to-shallow clay, clay loam, and sandy loam which support oak, juniper, water-tolerant hardwoods (Tuppad et al., 2010; USDA-SCS, 1986).

The Bosque watershed has a warm-temperate, sub-humid climate where the average annual precipitation ranges from about 737 mm to 838 mm, and daily mean temperature ranges from 36^o^F in January to 96^o^F in July (USDA NRCS, 2008). The environment of the Bosque watershed is characterized by high-intensity, short-duration rainfall events and other precipitation extreme events that can cause high surface runoff (USDA NRCS, 2008; Nielsen-Gammon et al., 2005). The winter and fall precipitation is induced by northern cold fronts associated with the Pacific winter storm, which produce low-intensity, long-duration storms (Wong et al., 2015). In the spring and summer, most precipitation produces high-intensity, short-duration storms that can result in flooding in small watersheds.

Several rivers and streams, such as Hico, Valley Mills, and Clifton, contribute to the Bosque River. Storm-driven runoff is a primary hydrologic event and source of water quality impairment in the North Bosque River (Mcfarland and Adams, 2020). Water pollution is a major water-related problem in the Bosque watershed. In 2000, this watershed was identified as an elevated concern of increased levels of nutrients entering the watershed from tributary watersheds. High levels of sediments, total nitrogen, and total phosphorus were identified (Tuppad et al., 2010).

### Data types and sources

The study has used spatial and non-spatial data acquired from different sources (Table 1). For climate change scenario development and SWAT model setup, climatic data such as precipitation and daily minimum and maximum air temperature (TMIN and TMAX) of all sub-basins of the Bosque watershed were obtained from Daymet gridded dataset. The Daymet is processed data largely derived from the stations’ data and includes remote sensing products to account for missing station data and spatiotemporal inconsistencies (Thornton et al., 2017). Daymet gridded observational dataset was also used to develop a baseline climate scenario (1981–2005) and for hydrological simulation during the baseline period. Daymet gridded dataset was selected because this dataset showed better performance than other gridded datasets in reproducing the in situ precipitation of the Bosque watershed (Ray et al., 2022). These climatic data were also used to generate weather station statistics needed to create SWAT’s weather generator input file (Arnold et al., 2012). The streamflow data of USGS discharge gauge stations located at different parts of the watershed were used. The USGS streamflow gauge stations are well distributed in the watershed, which increases the effectiveness of calibration as all of these stations are located at the outlets of sub-watersheds.

**Table 1:** Summary of data types, sources, and purpose used in the study

Dataset	Spatial resolution	Temporal resolution	Source	Purpose
DEM	30 m	–	Shuttle Radar Topographic Mission (SRTM)	Set up
Land Cover	30 m	–	National Land Cover Database (NLCD)	Set up
Soils	30 m	–	Gridded Soil Survey Geographic (gSSURGO)	Set up
Precipitation	10 km	Daily	Daymet	Set up
Temperature	10 km	Daily	Daymet	Set up
Streamflow	–	Daily	USGS discharge gauge stations	Calibration/validation
Climate model simulations	10 km	Daily	The South Central Climate Projections Evaluation Project (C-PrEP)	To develop future climate change scenarios

Spatial data such as land use, soil, and topography were required for hydrological modeling. The DEM data was used to create the basin boundary and stream networks. The slope generated from the DEM data was used to define Hydrological Response Units (HRU) and the soil and land use data. The Gridded Soil Survey Geographic (gSSURGO) was obtained from USDA’s Geospatial Data Gateway and used to set up the model. The land use/land cover data was obtained from the National Land Cover Database (NLCD). The latest iteration of NLCD, i.e., NLCD 2019, was used since this version contains 28 land covers characterizing land cover and change over different years from 2001 to 2019 (Dewitz and USGS, 2021). After identifying the different land use classes, they were redefined according to the SWAT land use database code. Thus, the land use/land cover of 2019 was used to represent the land use of the baseline period.

### Climate change scenario development

This study has used the South Central Climate Projections Evaluation Project (C-PrEP) dataset to develop future climate change scenarios. The C-PrEP has future projections of temperature and precipitation, which are produced from a combination of GCMs, emissions scenarios, downscaling techniques, and training data (Dixon et al., 2020). The GCMs used in the project are Community Climate System Model version 4 (CCSM4), Model for Interdisciplinary Research on Climate version 5 (MIROC5), and Max-Planck-Institute Earth System Model running on a low-resolution grid (MPI-ESM-LR). These GCMs were selected by C-PrEP based on their better performance in reproducing the historical precipitation and temperature of the south-central USA than other GCMs (Wootten et al., 2021).

The GCMs simulate the response of the 21^st^-century climate for three different future atmospheric composition and emission scenarios called Representative Concentration Pathways (RCPs 2.6, 4.5, and 8.5). The RCP8.5 represents high emission scenarios (radiative forcing pathway), resulting in 8.5W/m^2^ by 2100 (Van Vuuren et al., 2011a). The RCP4.5, which represents intermediate emission levels of 4.5 W/m^2^ that could start stabilization after 2100 (Moss et al., 2010), was used. The RCP2.6 emission scenario represents a very low emission scenario for the future. This emission scenario could only be achieved if the Paris Agreement and other sustainable and substantial measures to mitigate future climate change are realized.

The GCM simulations were downscaled using Daymet gridded observation dataset and different statistical downscaling techniques. The Ratio Delta method (DeltaSD) and the Equi-Distant Quantile Mapping (EDQM) are the statistical downscaling and bias correction techniques used to adjust GCM simulations. The future period used for climate scenarios was 2031–2099. A detailed description of GCMs, downscaling techniques, and Daymet datasets is given by Tefera et al. under review. Future climate change scenario development incorporates 3 GCMs, 2 statistical downscaling techniques, 3 emission scenarios, 1 gridded observation dataset, and three daily climate variables (maximum temperature, minimum temperature, and daily total precipitation).

### SWAT model setup

The hydrological model, SWAT, is used to predict the impact of land management and climate change on water, sediment, and agricultural chemical yields in large complex watersheds with varying soils, land use, and management conditions (Arnold et al., 1998; Gassman et al., 2007). The SWAT model is successfully applied in river basins and watersheds of Texas, USA, to study hydrological processes and other environmental applications such as the effect of land management and climate change on hydrology, water quality, and sediments (Chen et al., 2019, 2017; Elhassan et al., 2016; Stewart et al., 2006).

The SWAT model discretizes the Bosque watershed into 86 sub-basins. Further, the sub-basins were classified into Hydrological Response Units (HRUs). HRUs are unique combinations of slope, soil, and land use. Multiple HRUs were defined in a basin to allow heterogeneity within the basin. The HRU definition processes created 694 HRUs.

The surface runoff was estimated using the Soil Conservation Service (SCS), currently the Natural Resources Conservation Service, curve number computation (with modification) method. The Soil Conservation Service (SCS) Curve Number method is widely accepted to estimate surface runoff. The Penman-Monteith method was used to estimate potential evapotranspiration (PET) since it is a physical-based model.

### SWAT model calibration/validation and uncertainty analysis

**Fig. 2:**
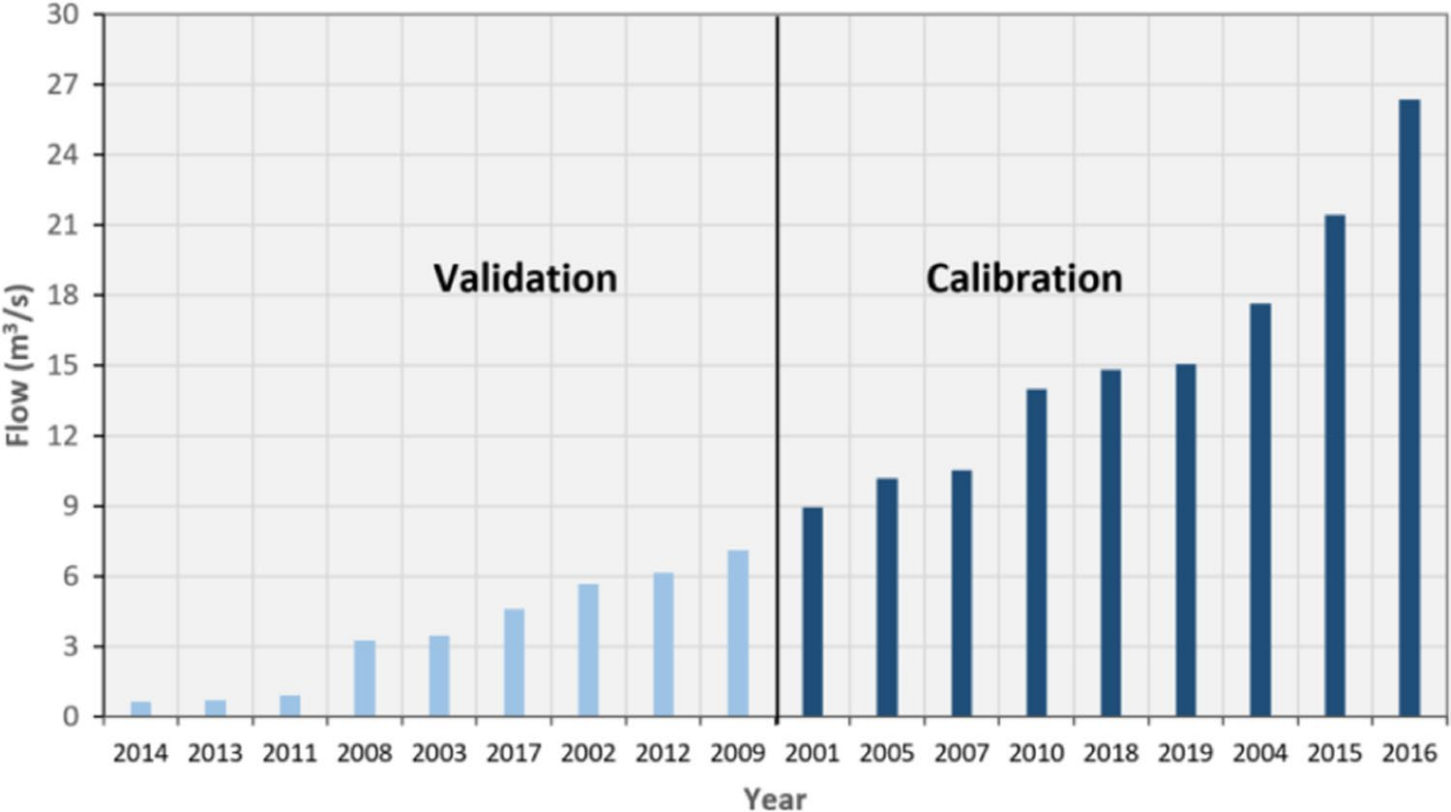
Observation years sorted based on streamflow (2001–2019) for Differential Split Sample Test (DSST). The flow is from the Valley Mills gauge; about 72% of the watershed drain toward this gauge

The SWAT model was calibrated and validated to represent the hydrologic conditions of the Bosque watershed before we used it to analyze climate change impacts. To robustly simulate the effect of climate change on hydrology, the SWAT model was calibrated and validated using the Differential Split Sample Test (Klemeš, 1986) method. The differential split sample calibration and validation approach is recommended whenever a hydrological model is intended to simulate the hydrology of watersheds under conditions different from the baseline conditions, such as climate change scenarios and land-use change studies (Daggupati et al., 2015). Accordingly, the years from 2000 to 2019 were grouped as dry and wet years based on their relative streamflow (Fig. 2). Since the simulation of different climate models under emission scenario projects a reduction of rainfall in the future period (section 3.1), the streamflow of wet years was used for model calibration, and the streamflow of dry years was used for validation at Valley Mills gauge of the Bosque watershed. Besides DSST approach, the model calibrated and validated using streamflow of wet and dry years was further validated at Crawford and McGregor gauges of the watershed. Thus, the calibration and validation were based on multi-site and DSST approaches.

The SUFI-2 in SWAT-CUP (Abbaspour et al., 2004) was used to perform sensitivity analysis, calibration, and validation of streamflow. Since there are many parameters in SWAT, only 12 parameters were identified as sensitive parameters through global sensitivity analysis. Parameters related to soil water, runoff, groundwater, evapotranspiration, and channel hydraulic conductivity were considered for the sensitivity analysis. The highest sensitive parameters with a smaller *p* value and larger t-test were selected (Abbaspour et al., 2015). Sensitive parameters were used to calibrate the streamflow of the watershed at different gauges (Table 2).

**Table 2:** Calibrated SWAT model parameters and parameter range using observed monthly streamflow at the Valley Mills gauge of the Bosque watershed. The model was calibrated using the streamflow of dry years from 2001 to 2019

No.	Parameter	Description of parameter	Min value	Max value	Fitted value
1	r__CN2.mgt	Curve number	−0.20	0.20	−0.11
2	v__ALPHA_BF.gw	Base-flow alpha factor (days)	0.00	1.00	0.44
3	v__GW_REVAP.gw	Groundwater “revap” coefficient	0.02	0.20	0.06
4	v__ESCO.hru	Soil evaporation compensation factor	0.40	1.0	0.98
5	v__CH_K2.rte	Effective hydraulic conductivity in main channel alluvium (mm/h)	5.00	130.0	91.4
6	v__ALPHA_BNK.rte	Base-flow alpha factor for bank storage (days)	0.00	1.00	0.80
7	v__SOL_AWC(..).sol	Available water capacity of the soil (mm)	0.00	1.00	0.46
8	v__REVAPMN.gw	Threshold depth of water in the shallow aquifer for “revap” or percolation to the deep aquifer to occur	0.00	500.0	119.8
9	v__SOL_BD(..).sol	Moist bulk density (Mg/m^3^ or g/cm^3^)	0.9	2.50	0.96
10	r__SOL_K(..).sol	Saturated hydraulic conductivity (mm/h)	−0.80	0.80	0.59
11	v__SURLAG.bsn	Surface runoff lag coefficient	0.05	24.0	21.96
12	v__GWQMN.gw	Threshold water depth in the shallow aquifer required for return flow to occur (mm)	0.00	500.0	1127.8

The qualifier (r_) refers to a relative change in the parameter where the default values are multiplied by 1 plus a factor in the parameter range, while (v_) refers to the substitution of the default parameter by a value from the parameter range. The extensions (e.g., .hru, .bsn, and .gw) indicate the SWAT parameter family

### Performance of calibration/validation and uncertainty analysis

**Fig. 3:**
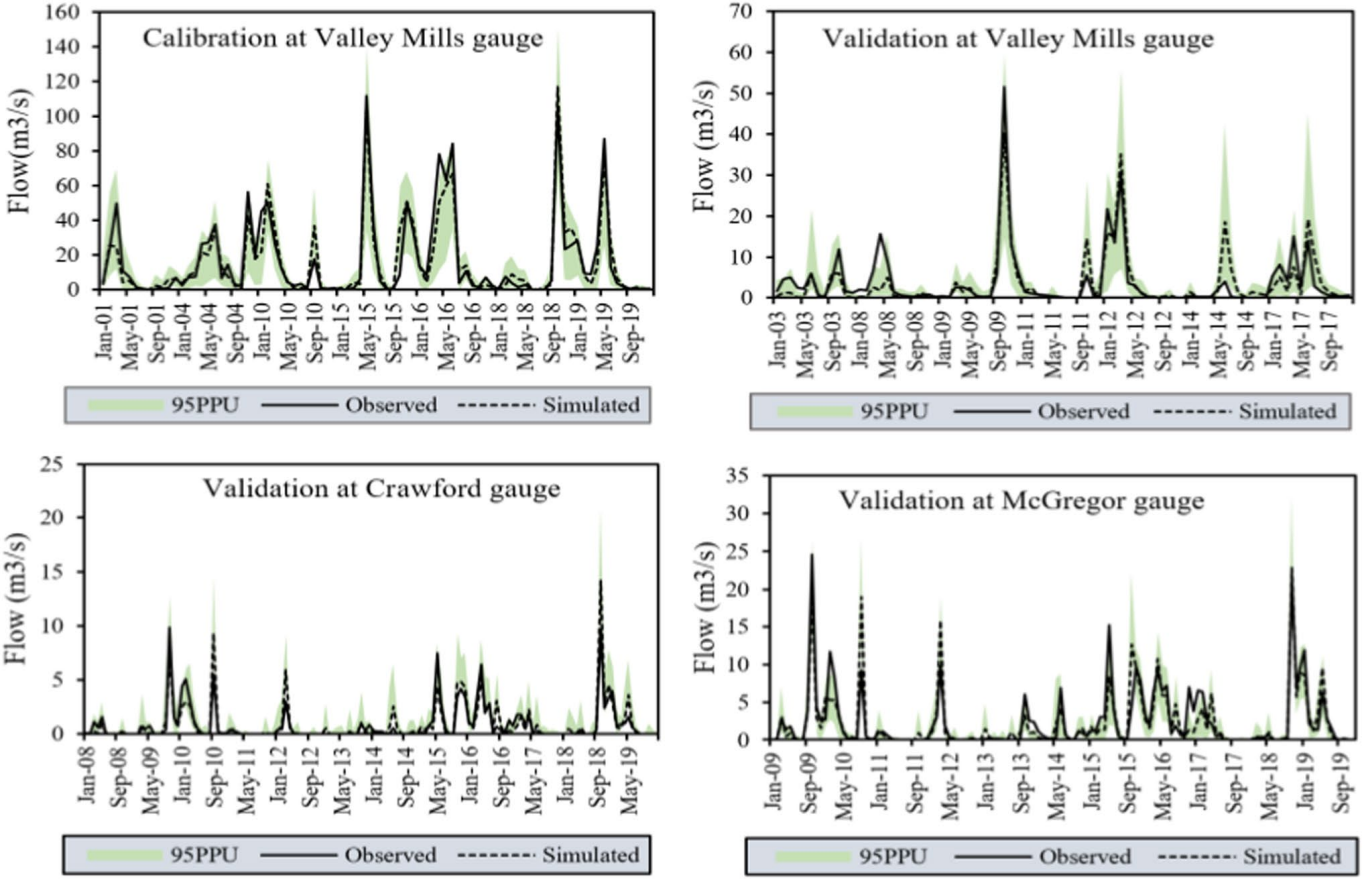
Simulated and validated hydrographs of calibration and validations at different gauges of the Bosque watershed

The performance of the hydrological model during calibration and validation at multi-gauges was evaluated using correlation coefficient (*R*^2^), Nash and Sutcliffe simulation efficiency (NSE), KGE (Kling–Gupta Efficiency) (Knoben et al., 2019), percent bias (PBIAS), and the ratio of the root mean square error to the standard deviation of measured data (RSR). These are important goodness-of-fit evaluation criteria (Moriasi et al., 2007; Moriasi et al., 2015; Abbaspour, 2015). The SWAT model calibrated and validated using these parameters and observed streamflow at all gauges revealed more than acceptable performance under different statistical metrics (Moriasi et al., 2015) (Table 3 and Fig. 3). The calibration at the Valley Mills gauge using the streamflow of the wet years showed very good efficiency (NSE = 0.89, KGE = 0.90, *R*^2^ = 0.90, and PBIAS = 4.5). The validation at the Valley Mills gauge using the streamflow of the dry years revealed NSE, *R*^2^, KGE, and PBIAS of 0.77, 0.78, 0.85, and 5.3, respectively (Table 3).

**Table 3:** Calibration and validation performance of the SWAT model at Valley Mills gauge of Bosque watershed

Objective functions	River stations			
	Valley Mills		Crawford	McGregor
	Calibration (wet years)	Validation (dry years)	Validation (2007–2019)	Validation (2008–2019)
*R*^2^	0.90	0.78	0.80	0.77
NSE	0.89	0.77	0.76	0.76
PBIAS	4.5	5.3	−3.4	3.4
KGE	0.90	0.85	0.85	0.87
P-factor (%)	87	77	65	67
R-factor	0.83	0.94	0.82	0.67

In most calibration and validation stages, the simulated hydrographs are well matched with the observed hydrographs (Fig. 3). However, in some cases, the model struggles to simulate the low and peak flows. For instance, at the Valley Mills gauge calibration, the model underestimated the peak flow in 2016 and 2019 (Fig. 3 and Table 3). All validations coherently underestimate the peak flow of 2009 at all gauges of the watersheds. Generally, there are more underestimations and overestimations at the Crawford gauge of the watershed than at other gauges.

Model calibration and validation were also evaluated using P-factor and the R-factor on the SUFI-2 algorithm (Abbaspour et al., 2004; Abbaspour et al., 2007). The SUFI-2 estimates uncertainty at 95 percent prediction uncertainty (95PPU). The P-factor is the percentage of the measured data bracketed within the 95PPU, while the R-factor measures the thickness of the uncertainty band. A P-factor of 1 and an R-factor of 0 indicate the exact fit of simulation with measurement (Abbaspour et al., 2007; Abbaspour, 2015). In most calibration and validation cases, the model achieved recommended P-factor value (>0.7) and recommended R-factor value (<1.5) (Abbaspour et al., 2015). The calibration at the Valley Mills gauge showed a P-factor of 87% and an R-factor of 0.83. Similarly, the validation using streamflow of dry years at this gauge also has a P-factor of 77% and an R-factor of 0.94. The validation at Crawford and McGregor gauges discloses a P-factor of 65 and 67 and an r-R-factor of 0.82 and 0.67, respectively (Table 3). The best P-factor and r-R-factor values were obtained at the Valley Mills gauge during the calibration period.

The SWAT model calibration and uncertainty analysis accentuate that the SWAT model can adequately simulate the streamflow of the Bosque watershed. The hydrological model is evaluated under changing climate conditions and multi-sites of the watershed. In future climate scenarios, precipitation reduction is projected in the study area. As a result, the model was calibrated using historical wet years and validated using historical dry years. This is because it is recommended to calibrate the hydrological model using DSST and wet years of the historical period (Daggupati et al., 2015; Krysanova et al., 2018). Thus, this model setup can be used to simulate climate change’s impact on the hydrology of the Bosque watershed. However, it is non-trivial to use robust climate scenarios developed from multiple climate model simulations, emission scenarios, and downscaling techniques to reduce uncertainty in climate change scenarios.

### Analysis of hydrology and hydrological extremes under climate change scenarios

The change in hydrological components, such as surface runoff, evapotranspiration, and streamflow under baseline and future climate change scenarios, was analyzed. For this, climate data of each climate scenario were used to force the SWAT model to be calibrated and validated using multi-gauge and DSST approaches (section 2.5). The hydrological extremes were analyzed using the mean discharge, Q5 (high flow), and Q95 (low flow). The Q5 and Q95 are commonly used flow indices and are defined as the flow values that exceed the flow time series data by 5% and 95% of the flow, respectively. Besides, the annual maximum and minimum flows (McMillan, 2021) were also used to analyze the extremes in streamflow under climate change scenarios. Thus, the changes in these hydrological extreme indicators between baseline and future climate change scenarios were analyzed.

## Result and analysis

### Climate change scenarios

**Fig. 4:**
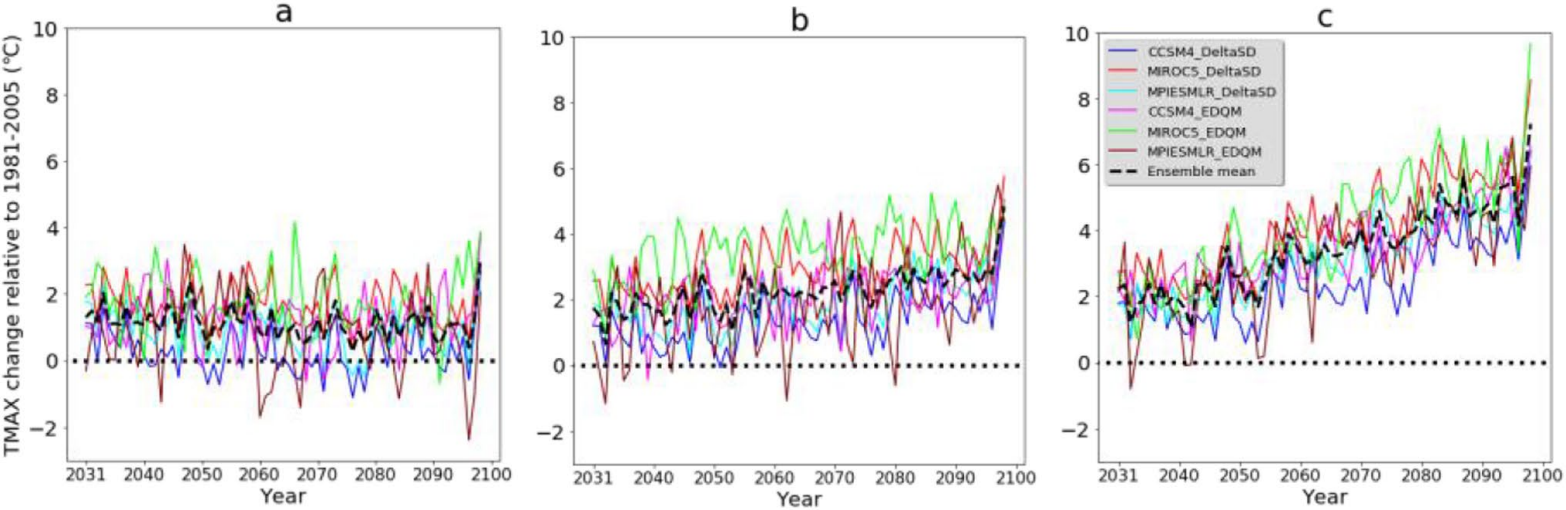

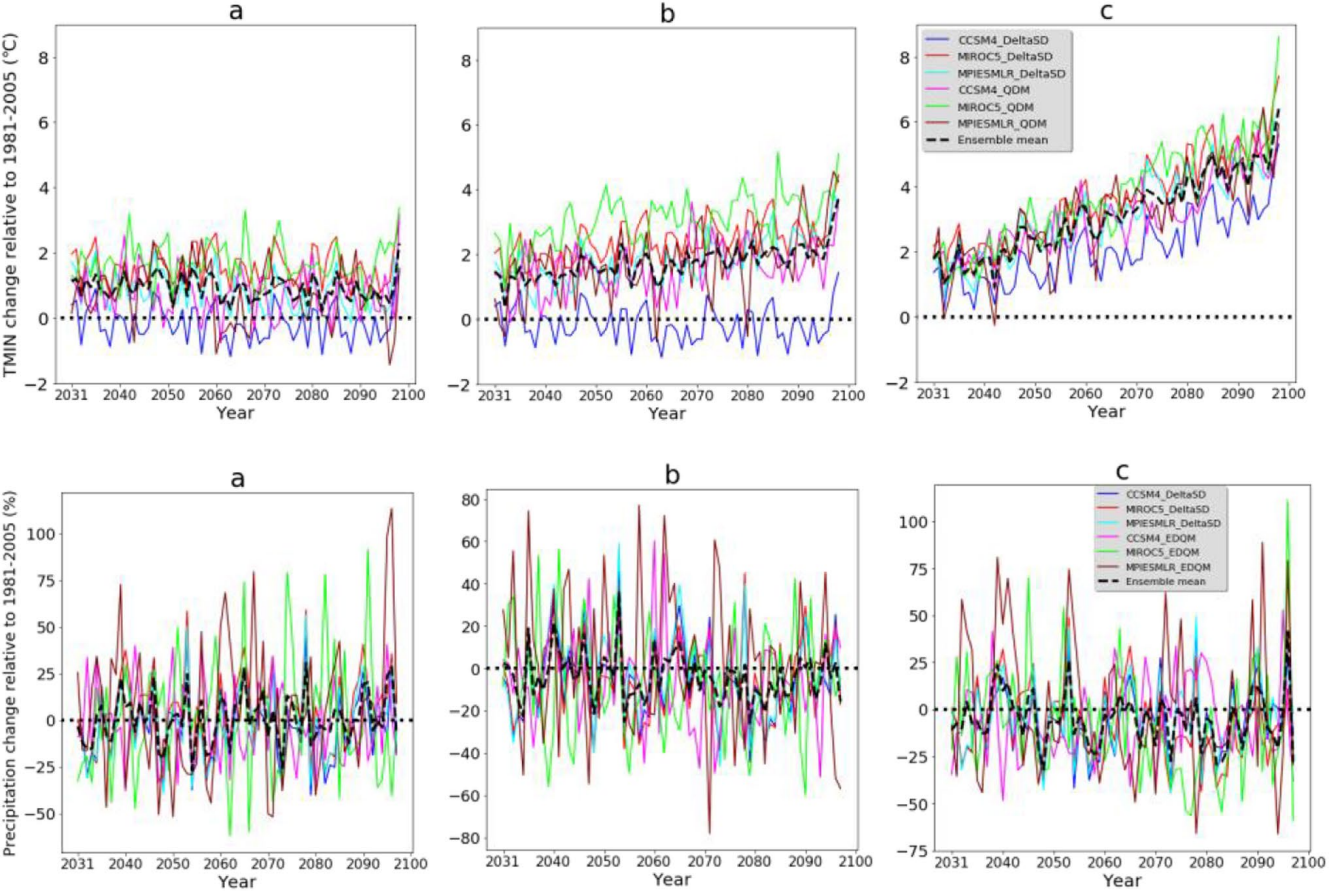
Changes in maximum and minimum temperature and precipitation in future climate change scenarios. **a** RCP2.6, **b** RCP4.5, and **c** RCP8.5 emission scenarios. DeltaSD and EDQM represent ratio delta and Equi-Distant Quantile Mapping statistical downscaling techniques, respectively

Most climate model simulations (S-RCMs) and ensemble mean of the simulations (E-RCMs) project a reduction of mean annual precipitation in the future and under all emission scenarios (Fig. 4). From the ensemble mean outputs, it is only under RCP2.6 an increase in precipitation is projected. In the future, the change in projected precipitation is in the range of −9.1% (MIROC5-EQDM under RCP8.5) to 4.9% (MPI-ESM-LR-EDQM under RCP2.6). A high decrease in precipitation is projected in the future and under RCP8.5 than other climate scenarios (Fig. 4). On the other hand, a high reduction of precipitation is also projected from GCM simulations downscaled by the DeltaSD method than the EDQM method. This indicates the choice of downscaling method has influenced the projected precipitation. In general, most future climate scenarios derived from different emission scenarios, GCM simulations, and downscaling techniques disclose similar future precipitation change signals and comparable rates of precipitation reduction.

Concurrent with these findings, projected precipitation showed variation following variation in emission scenarios in Texas High Plains (Chen et al., 2019). In the Texas High Plains, an increase in precipitation (in the order of 2.7% to 3.0%) under the RCP 2.6 emission scenario and a decrease in precipitation (in the order of 1.2% to 7.0%) under RCP 4.5 and RCP8.5 emission scenarios is projected for 2040–2099 (Chen et al., 2019). Similarly, a higher reduction (14%) of precipitation was projected in the 2090s under the high (A2) emission scenario in the Brazos River Basin of Texas (Awal et al., 2016). This coherence in the projection of precipitation among studies indicates a more likely decrease of precipitation in high and medium emission scenarios. Such precipitation changes may trigger tremendous environmental impacts, further affecting the water and agriculture sector.

Future climate change scenarios consistently project maximum (TMAX) and minimum (TMIN) temperatures. All future climate change scenarios revealed an increase in mean annual TMAX and TMIN in the future (Fig. 4). The increase in TMAX (TMIN) is in the range of 0.34 °C (−0.15 °C) (CCSM4-DeltaSD under RCP2.6) and 4.10°C (3.7°C) (MIROC5-DeltaSD and MIROC5-EDQM, respectively, under RCP8.5). However, the magnitude of change is different following the choice of emission scenarios, downscaling techniques, and climate models. The GCM simulations statistically downscaled by EDQM showed a higher increase of TMAX and TMIN than simulations downscaled by the DeltaSD downscaling technique. The ensemble mean of climate model simulations downscaled by DeltaSD and under the RCP8.5 emission scenario revealed an increase in TMAX (TMIN) of 3.27°C (2.96°C). In contrast, the ensemble mean of climate model simulations downscaled by EDQM and under the RCP8.5 emission scenario revealed an increase in TMAX (TMIN) of 3.56°C (3.29°C). The MIROC5 downscaled by DeltaSD and EDQM techniques showed a higher increase in TMAX and TMIN than other GCMs. Concurrent with the global temperature projection (IPCC, 2021, 2013), a higher increase in temperature is projected in the climate scenarios developed from the RCP8.5 emission scenario and the last decades of the 21^st^ century. Other studies in the Great Plains of Texas also project a consistent increase in temperature. For instance, in Texas, an increase in temperature (2.2–4.8°C) was projected relative to the mean of 1971−2000 from different climate model simulations and emission scenarios (Jiang and Yang, 2012). Such temperature changes could exacerbate existing environmental disasters such as wildfires which happened during the drought of 2011 and destroyed more than 1500 homes in Texas (USGCRP, 2018).

### Hydrological components under future climate change scenarios

**Fig. 5:**
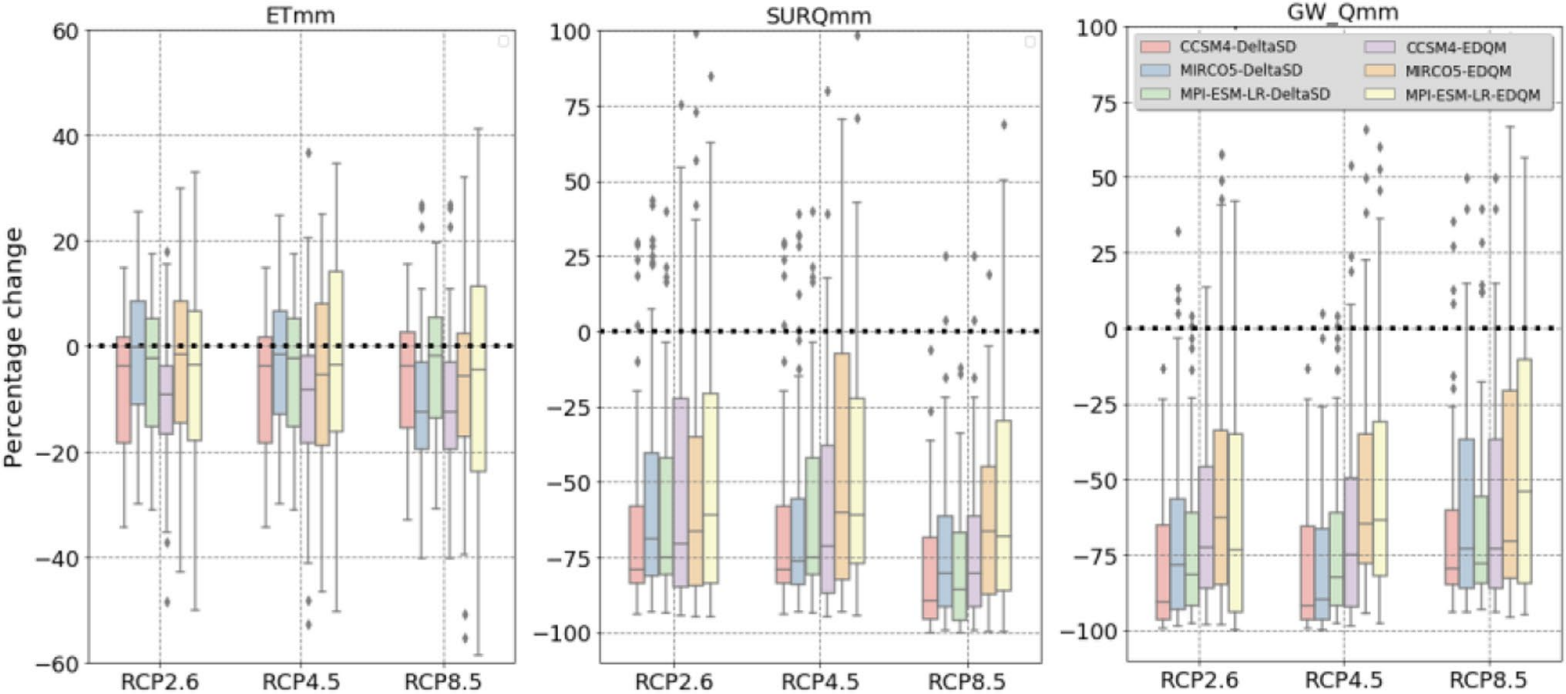
Net change (%) in hydrological components in future climate change scenarios from the historical period (2000–2019). ETmm = evapotranspiration in mm, SURQmm = surface runoff in mm, GW_Qmm = groundwater contribution to streamflow in mm

Hydrological components such as precipitation, surface runoff, groundwater, and water yield are projected to decrease in future climate scenarios. The reduction of surface runoff is in the order of 20% and 61%, while the reduction in groundwater is in the order of 27% and 59% (Fig. 5). Lower reduction of surface runoff, groundwater, and water yield is simulated from climate model simulations under RCP2.6 emission scenarios. However, no significant difference was observed in surface runoff, groundwater, and water yield simulated from climate model simulations under RCP4.5 and RCP8.5 emission scenarios revealed. Simulated hydrological components of future climate scenarios showed sensitivity to the choice of statistical downscaling techniques. Higher reduction of surface runoff, groundwater, and water yield is simulated from climate model simulations statistically downscaled by DeltaSD downscaling technique than EDQM downscaling technique. This is attributed to the difference in projected precipitation from DeltaSD and EDQM statistical downscaling techniques, where a higher precipitation reduction was projected in climate models downscaled by the DeltaSD downscaling technique (section 3.1).

The changes in simulated hydrological components are mainly attributed to the decrease in precipitation in future climate scenarios. Even the change in precipitation counterbalances the effect of temperature increase on evapotranspiration. Thus, some climate model simulations also simulate a reduction in evapotranspiration. Even though there is a higher increase in temperature in climate model simulations of RCP4.5 and RCP8.5, a negative change in simulated evapotranspiration is simulated in climate models under RCP4.5 and RCP8.5 emission scenarios. Comparatively, low temperature increases and lower precipitation reduction trigger an increase in simulated evapotranspiration in climate models under RCP2.6 emission scenarios.

**Fig. 6:**
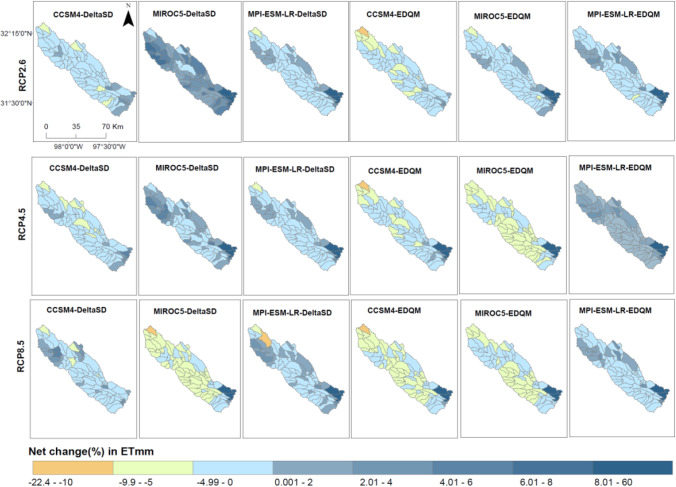

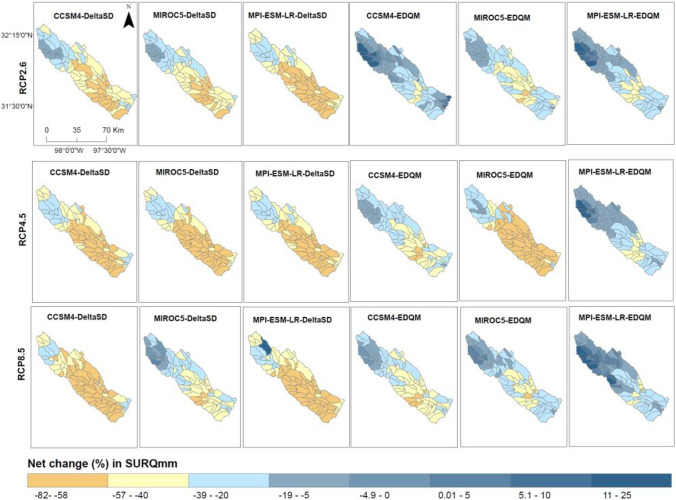
Net change in mean annual ETmm and SURQmm in the future climate scenarios. DeltaSD and EDQM represent ratio delta and Equi-Distant Quantile Mapping statistical downscaling techniques, respectively, and RCP represents Representative Concentration Pathways

Figure 6 presents the net change in evapotranspiration (ETmm) and surface runoff (SURQmm) under future climate change scenarios at the sub-basins of the Bosque watershed. A reduction in precipitation counterbalances a steady increase in temperature. Thus, a reduction in ETmm is simulated in most sub-basin and future climate change scenarios. The simulated ETmm and SURQmm showed variation following the driving GCMs and downscaling techniques. The simulations developed from the MPI-ESM-LR model revealed higher ETmm and SURQmm. In contrast, simulations in the MIROC5 GCM are characterized by a higher negative change in SURQmm and ETmm. Future SURQmm simulations in the EDQM downscaling techniques revealed a lower reduction of SURQmm. This is attributed to higher projected precipitation in the EDQM downscaling technique than in the DeltaSD downscaling technique.

Future ETmm and SURQmm also reveal variations among the sub-basin of the watershed. Projected higher precipitation in the southern part of the sub-basin resulted in an increase in ETmm in future climate change scenarios. However, an increase in evaporation in this sub-basin area further resulted in a significant reduction of SURQmm. The sub-basins in the southeastern part of the watershed simulate an increase in both ETmm and SURQmm. This accentuates an increase in precipitation which is available for evapotranspiration and SURQmm.

### Streamflow in future climate change scenarios

**Fig. 7:**
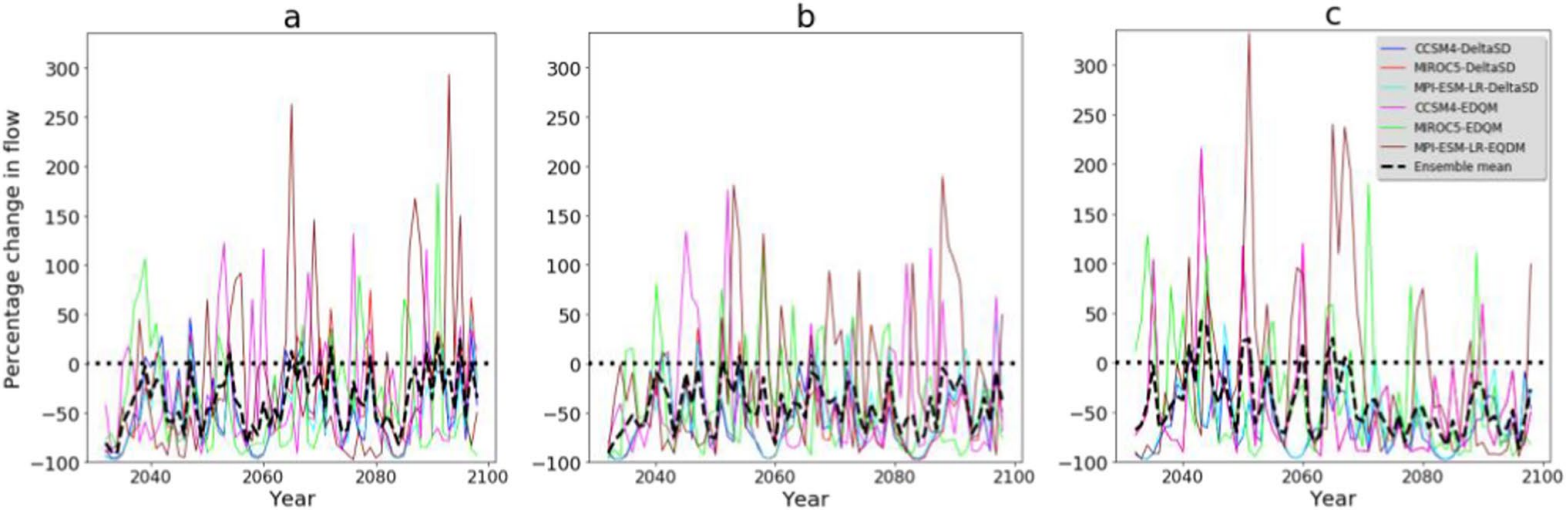
Percentage change of annual streamflow in future climate change scenarios. **a** RCP2.6, **b** RCP4.5, and **c** RCP8.5 emission scenarios. DeltaSD and EDQM represent ratio delta and Equi-Distant Quantile Mapping statistical downscaling techniques, respectively

The mean annual streamflow is projected to decrease consistently in future climate change scenarios. In the single climate model simulations, streamflow reduction is in the order of −14% (MPI-ESM-LR-EDQM) to −64% (CCSM4-DeltaSD). In contrast, the reduction in mean annual streamflow is in the range of −37% (−21% to −51%) in the climate model simulations under the RCP2.6 emission scenario and −43% (−17% to −58%) under the RCP4.5 emission scenario. Concurrent with future precipitation projection, future streamflow simulation shows sensitivity to the choice of statistical downscaling techniques and driving GCMs than emission scenarios. A higher reduction in streamflow is simulated in the GCM simulations downscaled by DeltaSD statistical downscaling technique than in GCM simulations downscaled by EDQM statistical downscaling technique (Fig. 7). Streamflow simulations derived from MPI-ESM-LR revealed a lower streamflow reduction, while streamflow simulations derived from MIROC5 revealed a higher streamflow reduction. In the RCP8.5 emission scenario, a higher streamflow reduction is simulated in the last three decades of the 21^st^ century (2070s to 2090s). Climate model simulations project an increase in streamflow in some years, but the ensemble mean under all emission scenarios reveals a consistent reduction of streamflow from 2031 to 2099 (Fig. 7).

**Fig. 8:**
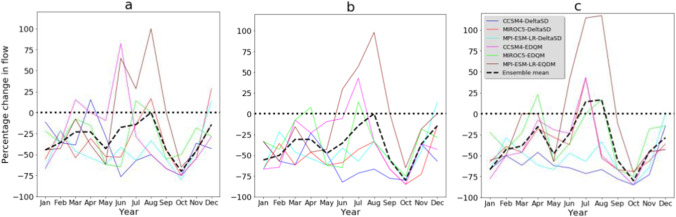
Percentage change of mean monthly streamflow in future climate change scenarios. **a** RCP2.6, **b** RCP4.5, and **c** RCP8.5 emission scenarios. DeltaSD and EDQM represent ratio delta and Equi-Distant Quantile Mapping statistical downscaling techniques, respectively

Parallel to the mean annual streamflow, the mean monthly streamflow is projected to decrease consistently in future climate change scenarios. Future streamflow simulation shows a change in a seasonal pattern where the wet months (May and October) in the baseline climate (Vogl and Lopes, 2009) are characterized by low streamflow, while dry months (July and August) in the baseline climate revealed lower reduction. Future streamflow in May shows a significant reduction (in the order of −10% to −67%). October, the second high streamflow season of the watershed, could face a significant reduction in streamflow in the future (Fig. 8). The ensemble mean of climate model simulations under the RCP8.5 emission scenario simulates an increase in streamflow in July and August (Fig. 8c). The choice of statistical downscaling techniques and driving GCMs influences future monthly streamflow simulations. The MPI-ESM-LR statistically downscaled by the EDQM technique simulates an increase in future streamflow in the June–September months (Fig. 8). In other studies, a streamflow reduction during 2040–2060 was also investigated in the Brazos River Basin, where the Bosque watershed is located (Wurbs et al., 2005). Even a negative trend in mean annual streamflow was observed at 10 Brazos River Basin gauge stations from 1955 to 2014 (Sohoulande Djebou, 2017). This reduction of streamflow in the study region will negatively impact different water uses such as irrigation water use, domestic water supply, and hydropower generation. Climate change already has a strong negative impact on water availability for irrigation on the Texas Rice Belt farmers and a reduction in instream flow needs in the Colorado River (USGCRP, 2018).

### Hydrological extremes in future climate change scenarios

**Fig. 9:**
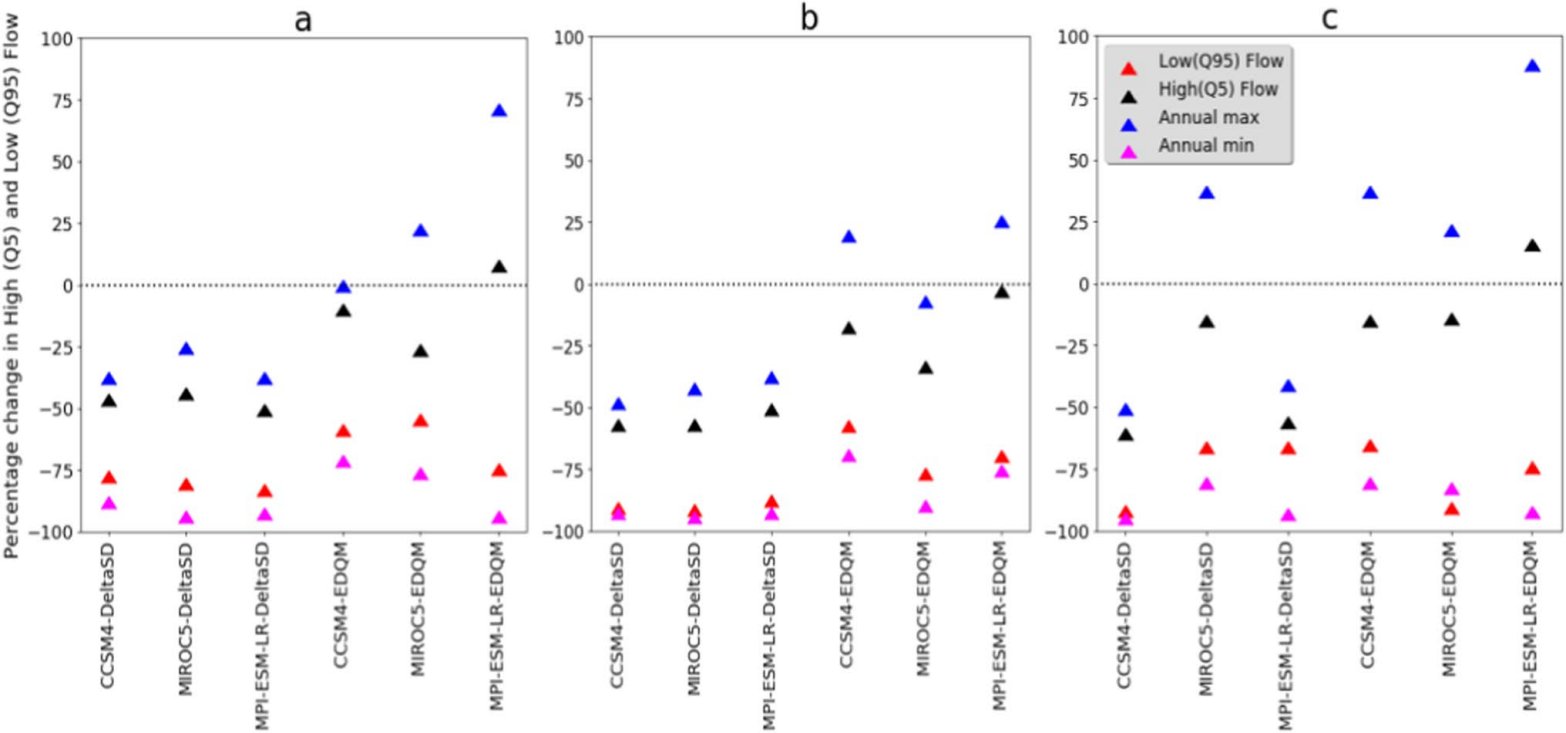
Percentage change in low flow (Q95), high flow (Q5), annual maximum flow, and annual minimum flow under future climate change scenarios. **a** RCP2.6, **b** RCP4.5, and **c** RCP8.5 emission scenarios. DeltaSD and EDQM represent ratio delta and Equi-Distant Quantile Mapping statistical downscaling techniques, respectively

The change in temperature and precipitation has resulted in a disproportional change in high flow (Q5) and low flow (Q95). Higher and consistent reduction of low flow is simulated in all climate scenarios (Table 4 and Fig. 9). The change in low flow is from −55% to −89%. A lower reduction of low flow (−57% to −86%) is estimated in the climate change scenarios developed from the RCP2.6 emission scenario. In comparison, a higher reduction of low flow (−55% to −89%) is simulated in the climate change scenarios developed from RCP4.5 emission scenario. The change in low flow also revealed a significant difference (*p*≤0.05) among the statistical downscaling techniques where higher low flow reduction is simulated in the GCMs downscaled by DeltaSD downscaling technique than the EDQM downscaling technique. However, the decline in high flow is lower than the low flow. Even an increase in high flow is estimated in some future climate change scenarios. The change in high flow is in the order of −59% to 18%. The percentage change in high flow is −22% in the ensemble mean of the RCP2.6 emission scenario, while it is −34% in the ensemble mean of the RCP4.5 emission scenario. Simulated high and low flows also show variation following the statistical downscaling techniques. For instance, in the climate model simulations downscaled by EDQM, the change in high flow ranges from −8% to 10%, −15% to −1%, and −10% to 18% under RCP2.6, RCP4.5, and RCP8.5 emission scenarios, respectively, while the change in high flow in the climate model simulations downscaled by DeltaSD is in the order of −44% to −49%, −48% to −55%, and −54% and −59% under RCP2.6, RCP4.5, and RCP8.5 emission scenarios, respectively.

**Table 4:** Percentage change in future streamflow characteristics under different climate model simulations

RCPs	Models	Annual	Annual max	Annual min	Q95	Q50	Q10	Q_5_
RCP2.6	CCSM4-DeltaSD	−43.89	−35.63	−85.98	−80.44	−32.72	−44.39	−44.22
	MIROC5-DeltaSD	−41.92	−23.08	−91.85	−83.24	−35.10	−40.25	−41.84
	MPI-ESM-LR-DeltaSD	−50.91	−35.26	−90.30	−85.55	−47.11	−49.02	−48.47
	CCSM4-EDQM	−28.57	2.06	−68.89	−61.26	−33.42	−29.60	−7.73
	MIROC5-EDQM	−35.62	24.50	−74.25	−57.20	−29.71	−32.50	−23.99
	MPI-ESM-LR-EDQM	−21.42	73.51	−91.69	−77.46	−32.26	−4.67	10.05
	Ensemble mean	−37.06	−44.26	−68.88	−32.02	−13.30	−49.62	−50.73
RCP4.5	CCSM4-DeltaSD	−57.70	−45.91	−90.36	−88.24	−52.35	−54.85	−54.68
	MIROC5-DeltaSD	−58.21	−40.16	−92.20	−89.21	−55.15	−60.27	−54.66
	MPI-ESM-LR-DeltaSD	−50.91	−35.26	−90.30	−85.55	−47.11	−49.02	−48.47
	CCSM4-EDQM	−35.26	21.82	−66.83	−55.18	−41.06	−25.00	−15.44
	MIROC5-EDQM	−40.01	−4.72	−87.60	−74.61	−39.56	−36.37	−31.08
	MPI-ESM-LR-EDQM	−16.89	27.50	−73.01	−67.19	−16.47	−6.91	−0.79
	Ensemble mean	−43.17	−52.67	−65.17	−19.59	−15.82	−57.24	−57.93
RCP8.5	CCSM4-DeltaSD	−63.75	−48.42	−92.33	−89.66	−61.34	−65.48	−58.52
	MIROC5-DeltaSD	−41.27	39.38	−78.37	−64.06	−43.37	−39.29	−12.90
	MPI-ESM-LR-DeltaSD	−56.64	−38.76	−90.77	−88.38	−51.48	−58.00	−53.65
	CCSM4-EDQM	−41.28	39.37	−78.40	−64.09	−43.37	−39.29	−12.90
	MIROC5-EDQM	−32.48	23.56	−80.50	−63.15	−33.01	−25.69	−11.82
	MPI-ESM-LR4-EDQM	−13.80	90.68	−89.79	−72.02	−21.93	−6.73	17.93
	Ensemble mean	−41.54	−37.41	−50.12	−26.02	−25.19	−47.84	−45.47

In future climate change scenarios, annual maximum and minimum flow revealed similar patterns with high and low flows, respectively (Fig. 9). All climate change scenarios simulated a higher reduction of annual minimum flow (−67% to −92%). However, simulated annual maximum flow showed a mixed trend where future climate change scenarios simulated an increase and a negative change in annual maximum flow. A change in annual maximum flow is in the order of −36% to 74%, −46% to 28%, and −48% to 91% under RCP2.6, RCP4.5, and RCP8.5 emission scenarios, respectively. This denotes that the streamflow of the Bosque watershed in the future will be characterized by extreme high and low flows, which could have multifarious effects on the natural ecosystems and the agricultural sector of the area. Similar to this study, a higher reduction of minimum flow than maximum flow was observed in 10 river gauges of the Brazos River Basin from 1955 to 2014 (Sohoulande Djebou, 2017). Projected hydrological extremes will increase the risks of existing hydro-climatic extreme impacts. Texas had several record-breaking floods in 2015, 2016, and 2017. The 2015 flood, even alone, has caused an estimated $2.6 billion in damage in Texas and Oklahoma (USGCRP, 2018).

**Fig. 10:**
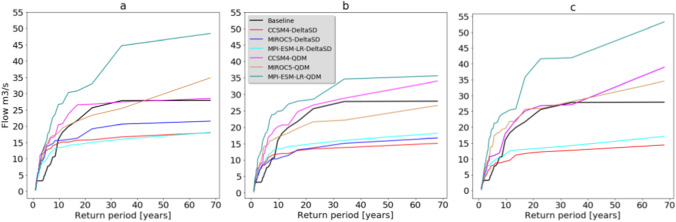
Annual flow return period of baseline and future climate change scenarios. **a** RCP2.6, **b** RCP4.5, and **c** RCP8.5 emission scenarios. DeltaSD and EDQM represent ratio delta and Equi-Distant Quantile Mapping statistical downscaling techniques, respectively

Despite the reduction in mean annual flow, the return period of high flow has revealed a mixed trend, whereas an increase in high flow is depicted in the return period of some future climate model simulations. The maximum flow in the baseline flow is lower than GCM simulations downscaled by EDQM statistical downscaling techniques (Fig. 10). The maximum flow occurring every 68 years is projected to increase from 28 m^3^/s in baseline climate to 42 m^3^/s in the GCM simulations downscaled by EDQM statistical downscaling techniques under the RCP8.5 emission scenario. The maximum flow occurring every 17 years is simulated to increase from 21 m^3^/s in baseline climate to 26 m^3^/s, 24 m^3^/s, and 29 m^3^/s in the GCM simulations downscaled by EDQM statistical downscaling techniques under RCP2.6, RCP4.5, and RCP8.5 emission scenarios, respectively. This indicates increased magnitude and frequency of high flow in some climate model simulations, while the maximum flow occurring every 68 years is projected to decrease from 28 m^3^/s in baseline climate to 23 m^3^/s in the GCM simulations downscaled by DeltaSD statistical downscaling techniques under the RCP8.5 emission scenario. The maximum flow occurring every 17 years is simulated to decrease from 21 m^3^/s in baseline climate to 15 m^3^/s, 13 m^3^/s, and 17 m^3^/s in the GCM simulations downscaled by EDQM statistical downscaling techniques under RCP2.6, RCP4.5, and RCP8.5 emission scenarios, respectively.

## Conclusion

The main objectives of this study were to quantify the changes in precipitation, temperature, surface runoff, evapotranspiration, and streamflow under future climate change scenarios and evaluate the magnitude of change in hydrological extremes under future climate change scenarios in the Bosque watershed, Brazos River Basin of Central Texas. The study showed how to integrate robust climate change scenarios with DSST and multi-site calibration and validation approaches. Using DSST and multi-site calibration and validation approaches, the study set up a hydrological model which shows more than acceptable performance (Moriasi et al., 2015). The parameters calibrated and validated at different gauges of the Bosque watershed can be used in other nearby watersheds with similar climate, topography, land use, and soil characteristics following the parameter transfer approach (Santhi et al., 2009).

Concurrent with the precipitation projection in the southern and southwestern regions of the United States (Romero-Lankao et al., 2014; USGCRP, 2018), most climate change scenarios in this study have negative signals indicating a future decrease in precipitation. The study also showed a steady increase in minimum and maximum temperature. The Great Plains region, including Texas, is under threat of different climate change impacts (Shafer et al., 2014). Projected changes in precipitation and temperature could escalate existing climate-driven impacts on the water and agriculture sector. This study also indicates significant variation in the magnitude of precipitation and temperature change following the choice of emission scenarios, GCM simulations, and downscaling techniques. For instance, GCM simulations downscaled by the DeltaSD technique showed a higher reduction of projected precipitation. However, most climate change scenarios derived from all downscaling techniques disclose negative precipitation change signals which corroborate a reduction in future precipitation, while GCM simulations downscaled by the EDQM technique showed a higher increase of projected TMAX and TMIN. Thus, climate change impact studies and climate adaptation decision analysis systems should consider such variations in climate change projections.

This study simulates a higher reduction of surface runoff, streamflow, and a negative change signal in evapotranspiration in future climate change scenarios. This is attributed to the compound effect of a reduction in precipitation and an increase in temperature. A higher surface runoff reduction is simulated in the climate model simulations developed from the RCP8.5 emission scenario and DeltaSD downscaling technique, which are characterized by a higher precipitation reduction and a higher temperature increase. A higher reduction in precipitation and a higher increase in temperature under the RCP8.5 emission scenario resulted in a negative change signal in evapotranspiration. The hydrological extremes in future climate change scenarios showed a steady decrease in low and annual minimum flows. This upholds there will be far lower flows in the dry seasons and years when drought occurs. However, an increase in high flow and annual maximum flow was simulated in some future climate change scenarios. For instance, a 10% and 18% increase in high flow was simulated in the MPI-ESM-LR4-EDQM under RCP2.6 and RCP8.5 emission scenarios, respectively. Unlike future trends in mean annual stream flow, annual maximum flow reveals an increase in most future climate change scenarios. Simulated hydrology and hydrological extremes revealed variation among the downscaling techniques. Parallel to precipitation projection, a higher reduction in surface runoff, groundwater, and streamflow was simulated in climate change scenarios derived from the DeltaSD downscaling technique than the EDQM counterparts. The variation in the influence of statistical downscaling techniques is strongly significant (*p*≤0.001) in the projection of high and low flows where far higher reductions of high flow and low flow follow the DeltaSD downscaling technique. Even climate change scenarios developed from EDQM downscaling technique project an increase in annual maximum flow. This corroborates a difference in the transfer functions in the DeltaSD and EDQM downscaling techniques to adjust extreme values.

These changes in hydrology and hydrological extremes could have multifarious effects on the water, agriculture, infrastructure, and other natural and anthropogenic systems. Central Texas and the Great Plains region, in general, are already characterized by recurrent drought, flood, and increase in drought severity (Rajsekhar et al., 2015; Shafer et al., 2014; USGCRP, 2018). The region is affected by a shortage of irrigation water (USGCRP, 2018). Thus, future changes in precipitation and evapotranspiration may escalate this problem by reducing soil moisture availability, irrigation water use, shortening the crops’ maturity period, and alterations in the hydro-ecosystems. Thus, water management structures, which can reduce the risk of flooding events, increase soil water availability, and non-structural water-based climate change adaptation decisions are essential in the watershed for the years to come. Similar to the current study, other studies that can provide better hydro-climatic information are central to building water management and the agriculture sector to buffer future hydro-climatic extremes.

## Data Availability

The datasets used or analyzed during the current study are available from the corresponding author upon reasonable request.
